# Formation of α-Farnesene in Tea (*Camellia sinensis*) Leaves Induced by Herbivore-Derived Wounding and Its Effect on Neighboring Tea Plants

**DOI:** 10.3390/ijms20174151

**Published:** 2019-08-25

**Authors:** Xuewen Wang, Lanting Zeng, Yinyin Liao, Jianlong Li, Jinchi Tang, Ziyin Yang

**Affiliations:** 1 Key Laboratory of South China Agricultural Plant Molecular Analysis and Genetic Improvement & Guangdong Provincial Key Laboratory of Applied Botany, South China Botanical Garden, Chinese Academy of Sciences, Xingke Road 723, Tianhe District, Guangzhou 510650, China; 2 University of Chinese Academy of Sciences, Yuquan Road 19A, Beijing 100049, China; 3 Center of Economic Botany, Core Botanical Gardens, Chinese Academy of Sciences, Xingke Road 723, Tianhe District, Guangzhou 510650, China; 4 Tea Research Institute, Guangdong Academy of Agricultural Sciences & Guangdong Provincial Key Laboratory of Tea Plant Resources Innovation and Utilization, Dafeng Road 6, Tianhe District, Guangzhou 510640, China

**Keywords:** aroma, *Camellis sinensis*, α-farnesene synthetase, herbivore, jasmonic acid, signaling, tea, volatile

## Abstract

Herbivore-induced plant volatiles (HIPVs) play important ecological roles in defense against stresses. In contrast to model plants, reports on HIPV formation and function in crops are limited. Tea (*Camellia sinensis*) is an important crop in China. α-Farnesene is a common HIPV produced in tea plants in response to different herbivore attacks. In this study, a *C. sinensis* α-farnesene synthase (CsAFS) was isolated, cloned, sequenced, and functionally characterized. The CsAFS recombinant protein produced in *Escherichia coli* was able to transform farnesyl diphosphate (FPP) into α-farnesene and also convert geranyl diphosphate (GPP) to β-ocimene *in vitro*. Furthermore, transient expression analysis in *Nicotiana benthamiana* plants indicated that CsAFS was located in the cytoplasm and could convert FPP to α-farnesene in plants. Wounding, to simulate herbivore damage, activated jasmonic acid (JA) formation, which significantly enhanced the *CsAFS* expression level and α-farnesene content. This suggested that herbivore-derived wounding induced α-farnesene formation in tea leaves. Furthermore, the emitted α-farnesene might act as a signal to activate antibacterial-related factors in neighboring undamaged tea leaves. This research advances our understanding of the formation and signaling roles of common HIPVs in crops such as tea plants.

## 1. Introduction

Tea (*Camellia sinensis*) plants are important woody economic crops with shoots and leaves that can be used to make the popular beverage [1]. Tea is usually grown in temperate and subtropical regions, such that insect attack is a normal annual phenomenon. In response to herbivore invasion, plants can release chemical compounds, known as herbivore-induced plant volatiles (HIPVs) [2,3]. HIPVs from economic crops, including tea plants, have been researched. Studies have indicated that (*Z*)-3-hexenol, nerolidol, (*E*)-β-ocimene, (*E*)-β-caryophyllene, (*E*,*E*)-α-farnesene and other terpenoids can be released when herbivores attack tea trees [4,5]. Furthermore, α-farnesene is a common volatile released by tea plants when suffering attack [6]. α-Farnesene has been reported to have an important effect on insect resistance, such as defense against of *Lobesia botrana* through locating spawning herbivores [7]. Compared with other economic plants, α-farnesene in tea plants has received little study.

The response to stress conditions, including insect resistance, of α-farnesene has been reported in several studies [8,9,10]. For instance, the functions of α-farnesene, linalool, and other inducible compounds are the same as (*Z*)-3-hexenol, which can be used by parasitoids to forage and locate hosts [11]. This indicates that many types of volatile can be considered as plant-to-plant and plant-to-herbivore communication signals [10,12]. A recent study reported that (*E*,*E*)-α-farnesene emitted by insect-induced soybean plays a defense role against nematodes [13]. Therefore, investigating the α-farnesene biosynthesis is of significance. α-Farnesene is classified as a sesquiterpene with coding genes that are part of or closely related to the plant terpene synthase gene (TPS) family [14,15]. The formation of monoterpene and sesquiterpene have been investigated in many plant species, including apple (*Malus domestica*) [16], *Arabidopsis* [17,18], soybean [13], and tomato [19]. (*E*,*E*)-α-Farnesene synthase has been found in apple fruit and expressed from the peel tissues [16]. However, the biosynthesis of α-farnesene in tea leaves has remained unknown. This study aimed to investigate the formation of α-farnesene in tea leaves exposed to herbivore attack and its potential signaling function.

## 2. Results 

### 2.1. Effect of Herbivore Attack on α-Farnesene Formation 

**Figure 1:**
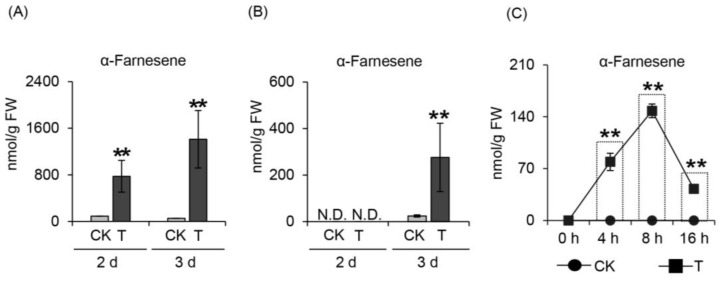
The formation of α-farnesene induced by two main herbivores and wounding stress in *C. sinensis* cv. Jinxuan. (**A**) Under tea green leafhopper attack; (**B**) Under tea geometrid attack. CK, without herbivores attack collected in different treatment time. T, with herbivores attack collected in different treatment time. (**C**) Under wounding stress; CK, without continuous wounding stress collected in different treatment time. T, with continuous wounding stress collected in different treatment time. Significant differences between control and treatment at the same treatment time are indicated (** *p* ≤ 0.01). N.D., not detected; FW, fresh weight.

Tea green leafhopper (*Empoasca* (*Matsumurasca*) *onukii* Matsuda) and tea geometrid (*Ectropis grisescens* Warren) are the two main tea plant herbivores and are classified as having piercing-sucking mouthparts and mandibulate mouthparts, respectively. When these two different species of herbivores separately attacked tea leaves of *C. sinensis* cv. Jinxuan, α-farnesene was detected in all groups over time. Furthermore, α-farnesene was produced in both insect treatments (Figure 1A,B). This showed that tea plants could release HIPVs, including α-farnesene, when attacked by different herbivore mouthparts. Although secretion from herbivore mouthparts could influence the HIPVs released to some extent, we concluded that wounding was the common factor during attack, and that α-farnesene produced by wounding might have an insect-resistance function. To verify this hypothesis, we mechanically damaged tea leaves to simulate herbivore attack and discovered the formation of α-farnesene with a significantly increased content compared with the control (Figure 1C). This result indicated that wounding stress that imitated herbivore attack could induce α-farnesene formation.

### 2.2. Identification of α-Farnesene Synthases in Tea Plants

**Figure 2:**
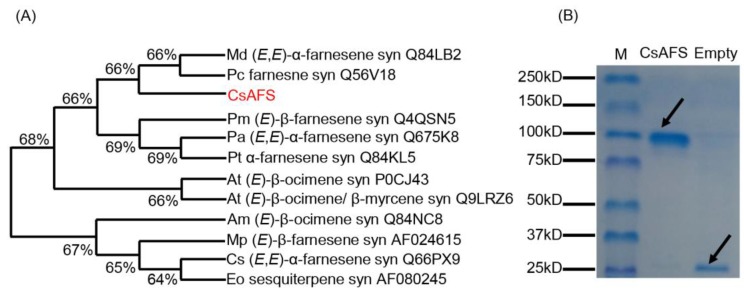
Phylogenetic analysis and SDS-PAGE analysis of α-farnesene synthase CsAFS. (**A**) The phylogenetic tree of α-farnesene synthase in different plant species, which was obtained by using MEGA-7 software with the neighbor-joining computation. Am, *Antirrhinum majus*; At, *Arabidopsis thaliana*; Cs, *Cucumis sativus*; Eo, *Elaeis oleifera*; Md, *Malus domestica*; Mp, *Mentha x piperita*; Pa, *Picea abies*; Pc, *Pyrus communis*; Pm, *Pseudotsuga menziesii*; Pt, *Pinus taeda*; CsAFS, α-farnesene synthase in *Camellia sinensis*. (**B**) SDS-PAGE analysis of recombinant CsAFS expressed in *Escherichia coli* (*E. coli*). Black arrows indicate target proteins.

**Figure 3:**
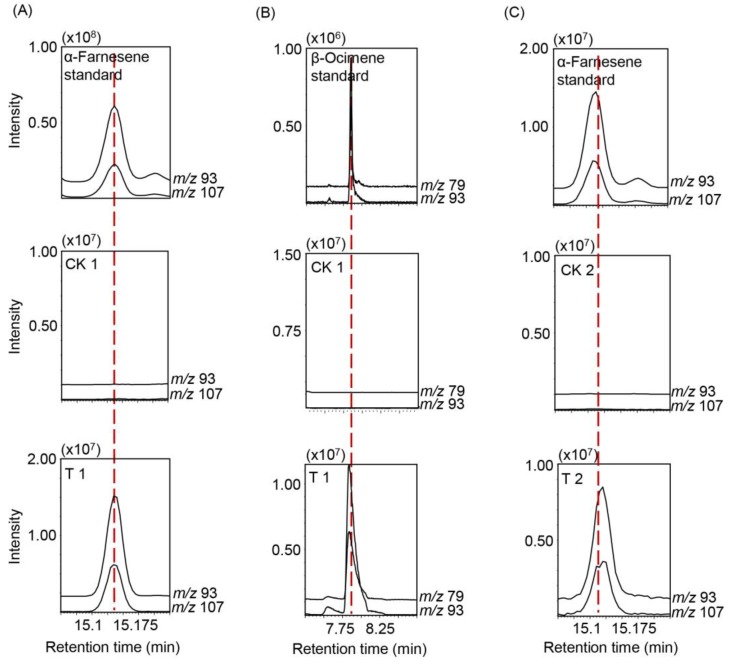
GC-MS analysis of the products formed by recombinant CsAFS enzyme *in vitro* and *in vivo*. (**A**) Functional identification of CsAFS *in vitro* with farnesyl diphosphate (FPP) as substrates; (**B**) Functional identification of CsAFS *in vitro* with GPP as substrates. CK 1, empty pET32a vector; T 1, recombinant CsAFS-pET32a protein. (**C**) Functional identification of CsAFS *in vivo*. The CsAFS-green fluorescent protein (GFP) fusion protein was transiently expressed in *N. benthamiana.* CK 2, empty GFP vector; T 2, recombinant CsAFS-GFP protein.

To identify the α-farnesene synthase (CsAFS) in tea plants, we compared homologous sequences of monoterpene and sesquiterpene synthases from the TPS family. Accordingly, we selected the syntheses of sesquiterpene, farnesene, α-farnesene, (*E*)-β-farnesene, (*E,E*)-α-farnesene, and (*E*)-β-ocimene in different plants belonging to the TPS-a, TPS-b, TPS-g, and TPS-d1 subfamilies (Figure 2A). Phylogenetic analysis indicated that CsAFS had a high similarity (66%) to MdAFS and farnesene synthase in *Pyrus communis* (pear) (Figure 2A), while only MdAFS in apple was related to (*E,E*)-α-farnesene formation. These results suggested that the *CsAFS* probably encoded α-farnesene synthase in tea plants. To validate the enzyme function of CsAFS in α-farnesene synthesis in tea leaves, cDNA of CsAFS was cloned into *Escherichia coli* (*E. coli*) expression vector pET32a, and the obtained recombinant protein was assayed for CsAFS function (Figure 2B). We used solid-phase microextraction (SPME) to collect products and utilized gas chromatography–mass spectrometry (GC-MS) to perform analysis. The results showed that recombinant protein CsAFS converted substrate farnesyl diphosphate (FPP) into α-farnesene as the final product. Furthermore, with geranyl diphosphate (GPP) as substrate, the CsAFS enzyme produced only β-ocimene, with no α-farnesene detected. No terpene products were found in the control, which carried the empty pET32a vector (Figure 3A,B). Our analysis of the recombinant CsAFS protein showed that the enzyme can produce α-farnesene or β-ocimene *in vitro* depending on the substrate. CsAFS enzyme activity produced α-farnesene as the only product from FPP as substrate.

**Figure 4:**
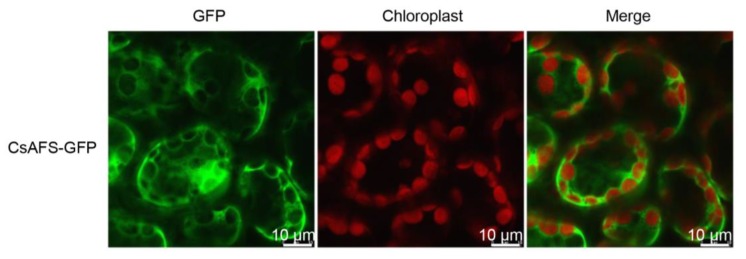
Subcellular localization of CsAFS protein in transformed *N. benthamiana*. Green fluorescence indicated CsAFS-GFP fusion protein, and red fluorescence indicated the chloroplast auto-fluorescence.

To further confirm the CsAFS function *in vivo*, we investigated the subcellular localization of CsAFS using green fluorescent protein (GFP)-fusion protein pCAMBIA3300. GFP analysis of epidermis cells from several independent tobacco plants transformed with CsAFS-GFP construct showed green fluorescence located in the cytoplasm, which clearly indicated that the CsAFS protein was targeted to the cytoplasm (Figure 4). GC-MS analysis of the products in transformed *Nicotiana benthamiana* showed that the CsAFS enzyme produced α-farnesene as a single product *in vivo* (Figure 3C). These findings supported that CsAFS probably used FPP as substrate to form α-farnesene in tea plants.

### 2.3. Effect of Wounding on CsAFS Expression, Jasmonic Acid Formation, and α-Farnesene Content in Tea Leaves

**Figure 5:**
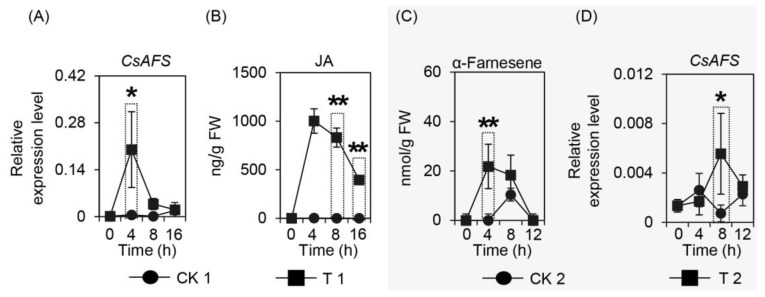
Changes in expression level of *CsAFS*, and contents of JA and α-farnesene under wounding stress and JA treatment, respectively. Variety of expression level of *CsAFS* (**A**) and JA content (**B**) after wounding stress. CK 1, without continuous wounding stress collected in different treatment time. T 1, with continuous wounding stress collected in different treatment time. Variety of α-farnesene content (**C**) and expression level of *CsAFS* (**D**) after JA treatment. CK 2, H_2_O containing 0.5% alcohol treatment collected in different treatment time. T 2, JA containing 0.5% alcohol treatment collected in different treatment time. Significant differences between control and treatment at the same different treatment time are indicated (* *p* ≤ 0.05, and ** *p* ≤ 0.01). JA, jasmonic acid; *AFS*, *α-farnesene synthase*; FW, fresh weight.

In a previous study, herbivore attack and wounding stress were the two main factors causing α-farnesene emission, with CsAFS enzyme located in the cytoplasm found to synthesize α-farnesene. To further confirm the relationship between wounding treatment and the α-farnesene synthesis gene, we analyzed *CsAFS* expression levels, with the results indicating that *CsAFS* was significantly upregulated after wounding treatment (Figure 5A). Furthermore, as jasmonic acid (JA) is an important signal factor in volatiles formation during mechanical damage, we also investigated changes in JA content under wounding treatment. The result showed that wounding stress had a significant effect on JA content (Figure 5B), and supported plants could produce JA when suffering mechanical damage. While further investigating the function of JA in α-farnesene synthase, the α-farnesene content was found to be significantly influenced by JA treatment (Figure 5C), and the *CsAFS* gene was also activated by JA treatment (Figure 5D). These results indicated the importance of JA in α-farnesene formation and provided a foundation to further investigate its function.

### 2.4. Effect of α-Farnesene on Neighboring Undamaged Tea Leaves

**Figure 6:**
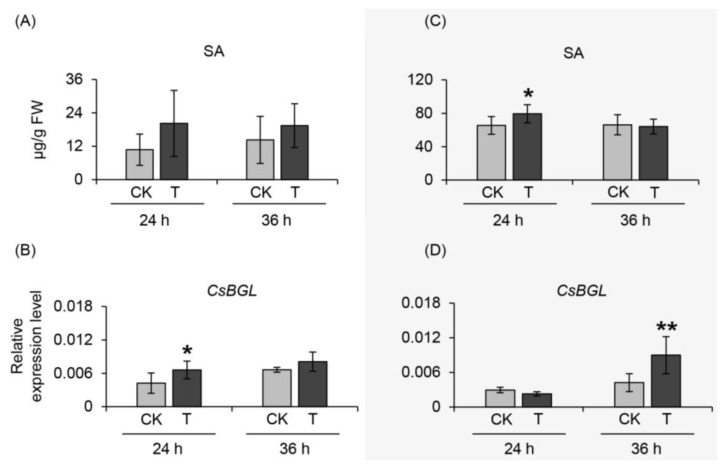
Changes in phytohormones content and expression levels of resistant related genes under α-farnesene in different treatment time, 24 h and 36 h. (**A**,**B**) All of the tea samples were collected in September 2018; (**C**,**D**) All of the tea samples were collected in June 2019. CK, dichloromethane (CH_2_Cl_2_) treatment collected in different treatment time. T, α-farnesene dissolving in CH_2_Cl_2_ treatment collected in different treatment time. Significant differences between control and treatment at the same treatment time are indicated (* *p* ≤ 0.05, and ** *p* ≤ 0.01). SA, salicylic acid; *BGL*, *β-1, 3-glucanase*; FW, fresh weight.

In this study, plant materials collected in September 2018 and June 2019 were treated with α-farnesene to explore its effect on neighboring undamaged tea leaves. Some phytohormones and antibacterial-related gene expression were investigated under these conditions. Analysis of the JA, salicylic acid (SA), and abscisic acid (ABA) levels showed that the JA and ABA contents did not significantly change in the materials collected at different times. In contrast, the SA content increased with α-farnesene treatment (Figure 6A,C). For ethylene (ET) analysis, we explored key synthetic genes of ET, namely *1-aminocyclopropane-1-carboxylate synthase* (*ACS*) and *ethylene-insensitive* (*EIN*) genes. No significant changes in gene expression levels were observed. Furthermore, we also investigated the genes related to resistance ability. The *β-1,3-glucanase* (BGL) gene expression level, which is related to antibacterial ability, was significantly increased with α-farnesene treatment (Figure 6B,D). These results improved our understanding of α-farnesene, which has potential functions in insect-resistant gene expression activation and as a signal to influence the defense ability of neighboring undamaged tea leaves.

## 3. Discussion

Herbivore attack is a common phenomenon in the process of plant growth and development, and plants can emit HIPVs in response to invasion [20,21,22]. Elicitors and mechanical damage are the main factors that induce HIPVs formation. Elicitors include fatty acid-amino conjugates (FACs) from herbivores’ oral secretion, hydrolase from herbivore saliva, and other compounds isolated from herbivores that react with plant cells, which can all induce specific production of HIPVs. Compound *N*-(17-hydroxylinolenoyl)-l-glutamine, which has been isolated from the oral secretions of beet armyworm caterpillars, can induce corn to emit volatile compounds similar to those emitted by corn under beet armyworm caterpillar attack when used on damaged leaves of corn [2]. Another report has indicated that *Heliothis virescens* and *Helicoverpa zea* can induce two different types of HIPVs when attacking cotton, corn, and tobacco [23]. Owing to their specificity, these types of HIPV could be candidates for narrow-spectrum insect resistance. In addition to elicitors, mechanical damage is a common factor resulting from all herbivore attacks. For example, when lima beans are subjected to continuous wounding treatment, the emitted volatiles are similar to those from lima beans exposed to *Spodoptera littoralis* attack [24]. During mechanical damage, phytohormones play an important role in volatiles formation, with the JA-mediated pathway considered the most important signal transduction pathway leading to HIPV emissions [25]. When suffering mechanical damage, plants can rapidly produce JA by activating its biosynthetic genes, and the synthetic JA can upregulate the formation of HIPVs through the downstream signal transduction networks [26,27,28]. In tea plants, mechanical damage can change the HIPV emissions significantly. After damage by *Tetranychus kanzawai,* tea geometrid, and tea green leafhopper, tea plants can release different levels of α-farnesene, (*E*)-3,8-dimethyl-1,4,7-nonatriene (DMNT) and (*E*)-β-ocimene [29,30]. Therefore, these HIPVs, including α-farnesene, resulting from wounding (Figure 1) might be broad-spectrum insect resistance candidates, which can be used for direct anti-insect compounds or attractants for insect enemies or plant defense-enhancing compounds.

Terpenoids are the most abundant class of natural products, showing structural diversity, including monoterpenes, diterpenoid, sesquiterpenoid, and triterpenoid and are the main components of HIPVs [31]. Many reports have confirmed that biotic and abiotic stresses can dramatically enhance most volatiles by regulating related genes important to the network, including terpene formation in tea plants [32,33,34,35,36]. α-Farnesene is a common volatile emitted by tea trees when suffering attack and has an important effect on insect resistance in many plant species. There are many researches reporting the genes on formation of farnesene or other terpenes in different plant species, while the α-farnesene synthesis genes with regards to tea plants have not been directly reported in recent studies. These genes coding terpene synthases belong to the TPS family, and are involved in both primary and secondary metabolism [37,38]. For example, (*E*)-β-ocimene synthase in *Antirrhinum majus* belongs to the TPS-g subfamily [39], (*E*)-β-farnesene synthase in *Mentha × piperita* belongs to the TPS-a subfamily [40], α-farnesene synthase in *Pinus taeda* and (*E,E*)-α-farnesene synthase in *Picea abies* belong to the TPS-d1 subfamily [38,41], and *MdAFS* coding (*E,E*)-α-farnesene synthase in *Malus domestica* belongs to the TPS-b subfamily [16]. To identify the α-farnesene synthase in tea plants, we compared these homologous sequences of monoterpenes and sesquiterpenes synthases among the TPS family. There is no successful genetic transformation system in tea plants, such that the evidence of metabolic pathways is difficult to obtain *in vivo*. Therefore, we used *N. benthamiana* as model plants to provide internal evidence. In our study, the results of *E. coli* expression *in vitro* indicated that the α-farnesene synthase, CsAFS protein, can produce α-farnesene or β-ocimene from different substrates (Figure 2 and Figure 3). For the different locations for monoterpene and sesquiterpene formation, we used subcellular localization to further confirm the functions potentially performed by this enzyme in tea plants (Figure 3 and Figure 4). These findings showed that α-farnesene synthase was an enzyme found in the cytoplasm, and that it was more likely to convert the substrate FPP into α-farnesene in tea plants. In the present study, we still could not exclude the possible occurrences of other *CsAFS* genes in tea because the expression of *CsAFS* was induced around 8 h after JA treatment, however, the accumulation of α-farnesene was detected around 4 h (Figure 5C,D). Future study on function validation of CsAFS (proposed in this study) using RNAi system in tea plants may find out if CsAFS has *in vivo* function in tea plants.

SA has an important effect on plant defense system activation after attack by pathogens [42] or herbivores [43,44] and the plant stress mechanism when confronting a poor environment [45,46]. Tobacco mosaic virus (TMV) has been reported to not only enhance the SA content in damaged leaves but also increase the endogenous SA levels [47]. These findings suggested that SA had a significant effect on plant defense signal transduction. Vernooij et al. indicated that SA was not responsible for the signal transduction to induce acquired resistance but was necessary in this translocation process [48]. Furthermore, exposure to β-ocimene has been indicated to arouse defense mechanisms in plants through SA, JA, and ET-mediated signaling pathways [49]. In plants suffering various stresses, it has been reported that ET is usually exposed and induced by 1-aminocyclopropane-1-carboxylate synthase (ACS) by improving the content of ET precursors [50,51]. Therefore, *ACS* and *EIN*, which belong to important genes involved in ET formation [52], were analyzed to determine the changes in ET. In this study, the SA content showed an increasing trend under α-farnesene treatment (Figure 6), while JA level and genes expression related to ET formation showed no significant changes, possibly due to antagonistic/synergistic cross-talk between phytohormone pathways [33]. Recently, a predominant isochorismate-derived biosynthesis of SA has been discovered in *Arabidopsis* plant. The key involved enzymes are isochorismate synthase 1 (ICS1), avrPphB susceptible 3 (PBS3), and enhanced disease susceptibility 5 (EDS5) [53]. It would be interesting to identify functions of these key involved enzymes in tea leaves and further investigate influence of α-farnesene treatment on them. β-Glucanases are considered important proteins for the response to fungal infection in pea endocarp. Sustained expression of the β-glucanase gene resulted in the general defense ability being enhanced by the enzyme [54]. Further evidence has shown that a shortage of β-1,3-glucanases can decrease the resistance to viral infections in mutant tobacco plants [55]. Similarly, in this study, expression of *CsBGL* was activated in tea plants under α-farnesene exposure (Figure 6). Although clear evidence of insect resistance was not obtained, these results provide some information showing that α-farnesene probably activates defense responses by influencing SA levels in neighboring tea plants.

## 4. Materials and Methods

### 4.1. Plant Materials and Treatments

Plant materials used in this study were sampled from the Tea Research Institute, Guangdong Academy of Agricultural Sciences (Yingde, China). The branches of *C. sinensis* cv. Jinxuan and Yinghong No. 9 plants, which contained the bud, first leaf, second leaf, and third leaf, were picked as experiment materials.

#### 4.1.1. Two Species of Herbivores Attack Treatment

Tea green leafhopper and tea geometrid, which are insects with piercing-sucking and mandibulate mouthparts, respectively, were used in this study. Each tea branch picked from *C. sinensis* cv. Jinxuan was placed in a plastic bottle containing five tea geometrids or twenty tea green leafhoppers to attack the tea branches. The treatment conditions were 28 °C with 80% humidity for 48 h and 72 h. The control groups without attacking insects were treated under the same conditions. All samples were then frozen in liquid nitrogen and kept at −80 °C for further study.

#### 4.1.2. Continuous Wounding Treatment

As insect attack is a continuous wounding process. It is difficult to precisely simulate insect attack pattern, wounding intensity, and frequency. Here, a shaking table (HZ-9310KBG, Hualida, Jiangsu, China) was used to simulate wounding stress. It can keep the picked tea leaves continuously exposed to wounding treatment [56,57,58,59], although it cannot precisely simulate insect attack. We put the picked tea leaves of *C. sinensis* cv. Jinxuan in the shaking table at 200 rpm and 25 °C. The control groups were placed in the same conditions without shaking. Afterward, all samples were collected at 0, 4, 8, 16 h, frozen with liquid nitrogen, and kept at −80 °C for further study.

#### 4.1.3. JA Treatment

We picked branches from *C. sinensis* cv. Jinxuan to place in JA solution (2.5 mM, containing 0.5% alcohol). Samples were kept at 25 °C, cultured in a 12-h light/12-h dark cycle, and the humidity was 80%. The control groups were only treated with H_2_O containing 0.5% alcohol under the same conditions. After treatment, samples were collected at 0, 4, 8, 12 h, frozen with liquid nitrogen, and kept at −80 °C for further study.

#### 4.1.4. α-Farnesene Treatment

The plant materials picked from *C. sinensis* cv. Yinghong No. 9 were placed in a closed jar containing a cotton ball. α-Farnesene standard (500 µL, 4 mM) in dichloromethane (CH_2_Cl_2_) was injected into the cotton ball every 12 h as treatment. The total α-farnesene content in the treatment was 6 µmol. In the control groups, CH_2_Cl_2_ was injected into the cotton ball instead of the α-farnesene standard. The total volume of CH_2_Cl_2_ in the control was 1.5 mL. Samples were collected at 24 h and 36 h, frozen with liquid nitrogen, and kept at −80 °C for further study. As α-farnesene is water insoluble, CH_2_Cl_2_ was used as solvent to dissolve the α-farnesene standard to achieve fumigation. A previous report indicated that the influence of CH_2_Cl_2_ was the same as that of H_2_O, with no negative effects [60].

### 4.2. Analysis of α-Farnesene Content in Tea Leaves

In herbivores attack treatment and wounding treatment, 200 mg finely powdered fresh tea leaves of each sample was extracted with 700 μL CH_2_Cl_2_ containing 2 nmol ethyl decanoate as an internal standard. In JA treatment, we adopted 1.8 mL CH_2_Cl_2_ containing 5 nmol ethyl decanoate which was considered as internal standard, to extract 500 mg finely powdered fresh tea leaves of each sample. Afterward, all samples were shaken for 5 h at 25 °C. The filtered extraction solution was dried with anhydrous Na_2_SO_4_ to remove water and evaporated under nitrogen flow until had a 100 μL extract. Finally, samples were analyzed by GC-MS (specific method and details of GC-MS are provided in the Supplementary Materials).

### 4.3. Analysis of Phytohormones Content in Tea Leaves

In wounding treatment and α-farnesene treatment, we used 2 mL ethyl acetate to extract 200 mg finely powdered fresh tea leaves of each sample. This was followed by vortexing for 30 s and adding [^2^H_5_] JA to the mixture as an internal standard in continuous wounding treatment, while adding [^2^H_4_] SA, [^2^H_5_] JA and [^2^H_6_] ABA to the mixture as internal standards in α-farnesene treatment. Afterward, all samples were put in ice-cold water with ultrasound for 20 min, and the supernatants were collected through centrifuging at 10,000× *g* for 5 min at 4 °C. Then all extraction solutions were dried under nitrogen flow, redissolved in methanol (200 µL), and filtered through a 0.22 µm membrane. Finally, samples were analyzed by an ultra-performance liquid chromatography/quadrupole time-of-flight mass spectrometry (UPLC-QTOF-MS) (specific method and details are provided in the Supplementary Materials).

### 4.4. CsAFSs Recombinant Expression and Enzyme Assay

#### 4.4.1. RNA Isolation and Reverse Transcription

The RNA used in this study was isolated from finely powdered fresh tea leaves using a Quick RNA Isolation Kit (Huayueyang, Beijing, China), while cDNA was obtained using a Prime Script RT reagent Kit with gDNA Eraser (Takara, Dalian, China) to complete reverse transcription from RNA.

#### 4.4.2. Gene Cloning

The *CsAFS* gene sequence in tea plants (Accession number: GFMV01032657) was identified through homologous alignment with terpene synthesis-related genes based on the phylogenetic tree, which was prepared using MEGA-7 software with the neighbor-joining computation. The *CsAFS* gene was obtained by PCR using an In-Fusion HD Cloning Kit (Takara Biotechnology, Shiga, Japan). Two rounds of PCR primers, designed by Primer Premier 6, are listed in Table 1. The first-round reaction was performed as follows: 98 °C for 3 min; 33 cycles of 98 °C for 30 s, 60 °C for 10 s, 72 °C for 30 s, and 72 °C for 10 min; and then kept at 16 °C. The second-round reaction was performed as follows: 98 °C for 3 min; two cycles of 98 °C for 30 s, 45 °C for 10 s, and 72 °C for 30 s; 33 cycles of 98 °C for 30 s, 66 °C for 10 s, and 72 °C for 30 s; and then kept at 16 °C. The final purified cloned products were obtained using a Gel Extraction Kit (Huayueyang, Beijing, China), then cloned into pET32a vector (Novagen, Madison, WI, USA), and transformed into DH5α competent cells (Novagen, Madison, WI, USA) for sequencing.

#### 4.4.3. *E. coli* Expression of CsAFS

We transformed the expression vector into *E. coli* Rosetta cells (Novagen, Madison, WI, USA) for protein expression. Firstly, transformed Rosetta cells with a recombinant vector (100 mL) were incubated at 37 °C and 200 rpm until the OD_600_ was 0.5. Next, 0.1 mM IPTG was added to the bacterial fluid, which was then kept at 18 °C and 200 rpm for 16 h, and finally cells were collected after centrifugation at 10,000× *g* for 5 min at 4 °C. After resuspending the precipitate in 25 mM, pH 7.4 Tris-HCl buffer, the mixture was disrupted by sonication for 15 min and then centrifuged at 10,000× *g* for 10 min at 4 °C. The supernatant was collected and filtered. Ni-NTA bind resin (GE Healthcare, Uppsala, Sweden) was used to purify the supernatant, and the partially purified protein was then purified through a PD-10 desalting column (GE Healthcare, Buckinghamshire, UK). The protein was finally eluted with Tris-HCl (25 mM, pH 7.4) for further study.

#### 4.4.4. SDS-PAGE Analysis of CsAFS

SDS-PAGE uses a separation gel and concentration gel to perform electrophoresis and colloidal staining methods to achieve protein separation. Afterwards, they were stained with Coomassie Blue Fast Staining Solution (R250) for 30 min and then decolored three times by water.

#### 4.4.5. Enzyme Assay and Functional Characterization of CsAFS

Two different substrates, FPP and GPP (Sigma Aldrich, St Louis, MO, USA), were separately reacted with CsAFS protein (15 μg) in buffer (1 mL). pET32a empty vector protein was treated under the same conditions as control. All reactions were performed in bottles sealed using parafilm. The reaction was performed at 30 °C for 1 h, and α-farnesene was collected by solid-phase microextraction (SPME) (2 cm, 50/30 μm, DVB/Carboxen/PDMS Stable Flex, Bellefonte, PA, USA) during this time. The enzyme assay buffer was composed of 10 mM Na_2_HPO_4_, 1.8 mM NaH_2_PO_4_ (pH 7.3), 140 mM NaCl, 10 mM MgCl_2_, 5 mM dithiothreitol, 500 mM KCl, 1 mM MnCl_2_, 0.05% (*w/v*) NaHSO_3_, and 10% (*v/v*) glycerol, adjusted to the optimum pH 7.0 [16]. Volatiles absorbed by SPME and the α-farnesene standard (Sigma Aldrich, USA) dissolved in CH_2_Cl_2_ were subjected to GC-MS analysis (using the method described in the Supplementary Materials).

### 4.5. Subcellular Location Analysis of CsAFS

The *CsAFS* gene was cloned and inserted into pCAMBIA3300-GFP vector using an In-Fusion HD Cloning Kit (Takara Biotechnology, Japan). The primers designed by Primer Premier 6 are listed in Table 1. The recombinant vector was then transformed into *Agrobacterium* GV3101 (Novagen, Madison, WI, USA). A single colony was selected and transferred into Luria-Bertani (LB) medium (5 mL) containing Rif, Gen, and Kana antibiotics and kept overnight at 28 °C. Next, 1 mL of the overnight culture was added into LB medium (50 mL) containing 20 μM acetosyringone and again kept overnight at 28 °C. The sample was then centrifuged at 5000× *g* for 5 min, and the precipitate was resuspended in resuspension buffer containing 10 mM MgCl_2_, 10 mM morpholineethane-sulfonic acid (MES-K, pH 5.6), and 100 mM acetosyringone until the OD_600_ was 0.4. Cells were placed at 25 °C without shaking for 2 h. A needleless syringe was used to infiltrate GV3101 containing pCAMBIA3300-CsAFS into two-to-four-week-old *N*. *benthamiana* leaves. The pCAMBIA3300 vector was used as control. The tobacco plants were cultured in a 12-h light/12-h dark cycle at 25 °C for 4–5 days after infiltration. After growing, infiltrated tobacco leaves were immersed in water (40×) with an excitation wavelength of 488 nm for GFP fluorescence observation (Leica TCS SP8 STED 3×, Mannheim, Germany).

### 4.6. Analysis of CsAFS Activity in N. Benthamiana Overexpression Lines

After subcellular location analysis, the infiltrated fresh tobacco leaves were frozen with liquid nitrogen and kept at −80 °C for further study. We added 1.8 mL CH_2_Cl_2_ to extract 300 mg finely powdered fresh tea leaves followed by mixing 5 nmol ethyl decanoate as internal standard. Then all the samples were extracted at 25 °C for 5 hours. Anhydrous Na_2_SO_4_ was used to remove water in extraction solution that was then concentrated using nitrogen flow. The final 200 μL extracts and the α-farnesene standard dissolved in CH_2_Cl_2_ were then subjected to a GC-MS analysis (using the method described in the Supplementary Materials).

### 4.7. Transcript Expression Analysis of Genes

The gene expression levels were analyzed using the RNA isolation and reverse transcription methods described in Section 4.4. Quantitative real-time PCR (RT-qPCR) was conducted using a Roche LightCycler 480 system (Roche Applied Science, Mannheim, Germany). In this study, changes in the target genes were compared with the internal reference gene, encoding elongation factor 1 (*EF1*). All specific primers are listed in Table 2. The specific method and details of RT-qPCR are provided in the Supplementary Materials.

### 4.8. Statistical Analysis

Excel Ver. 2013 software was used to achieve statistical analysis. The significant differences between two treatment groups were determined by a two-tailed Student’s *t*-test. *p* ≤ 0.05 was considered as significant.

## 5. Conclusions

**Figure 7:**
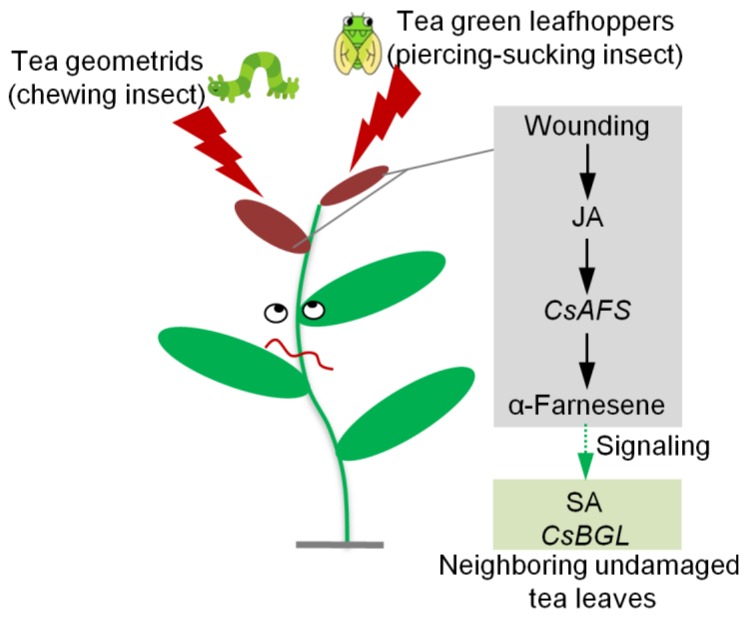
Summary on herbivore-induced formation of α-farnesene and its effect on defense ability of neighboring tea plants as a volatile signal. JA, jasmonic acid; *AFS*, *α-farnesene synthase*; SA, salicylic acid; *BGL*, *β-1, 3-glucanase*.

In this study, α-farnesene was found to be a common HIPV in tea leaves. CsAFS was validated to have a function in α-farnesene synthesis and was found to be located in the cytoplasm using *E. coli* expression analysis, *N. benthamiana* expression analysis, and subcellular localization. Wounding, a common outcome of herbivore attack, activated JA formation. JA significantly enhanced the CsAFS expression level, which led to the biosynthesis of α-farnesene. Furthermore, emitted α-farnesene might act as a signal to activate antibacterial activity in neighboring undamaged tea leaves (Figure 7). This information will contribute to our understanding of HIPV formation in tea plants and provide metabolite candidates for applications in tea plant defense against environmental stresses.

## Figures and Tables

**Table 1:** Primers used for cloning *CsAFS* and subcellular location.

**Primers Used for Cloning *CsAFS*-pET32a**	
*CsAFS*-Forward	CCACAACTTTCCCACCTCTTCTT
*CsAFS*-Reverse	CAAATTATTTGGTGAGACCTGTGGAGG
*CsAFS*-*EcoRV*-Forward	AAGGCCATGGCTGATATCATGGATTGTAGTAAAGGAATGCTAG
*CsAFS*-*SalI*-Reverse	CCGCAAGCTTGTCGACTCAATTGATCTTGAGAGGTTCG
**Primers Used for Cloning *CsAFS*-GFP**	
*CsAFS*-Forward	CCACAACTTTCCCACCTCTTCTT
*CsAFS*-Reverse	CAAATTATTTGGTGAGACCTGTGGAGG
*CsAFS*-*SacI*-Forward	AGAACACGGGGGACGAGCTCATGGATTGTAGTAAAGGAATGCTAG
*CsAFS*-*SpeI*-Reverse	CCATGGTGGCACTAGTATTGATCTTGAGAGGTTCGATAAGC

**Table 2:** Primers used for RT-qPCR analysis of genes involved in synthesis of α-farnesene and defense ability in tea leaves.

Gene Name	Accession Number	Forward Primer 5′–3′	Reverse Primer 5′–3′
*CsEF1*	KA280301	TTGGACAAGCTCAAGGCTGAACG	ATGGCCAGGAGCATCAAT GACAGT
*CsAFS*	GFMV01032657	TGTCAACACAGCTAGAGTGGC	AGCATAGAGAGGACTTGGGC
*CsACS*	-	TGAGAGGCGATAGAGTGACATT	GCTGCTTCCAAGGCTGATT
*CsEIN3*	-	GGTAAGGAAGGAGTTGATGCT	CCGCTTGGTATTTCGCTATTG
*CsBGL*	GGEB01035404	GTGCCCCATTGCTCCTTAAC	TTCCACCAACTGTAGGCCAT

EF1, encoding elongation factor 1; ACS, 1-aminocyclopropane-1-carboxylate synthase; EIN3, ethylene insensitive protein 3; BGL, β-1, 3-glucanase. ‘-’, the accession number of gene has not been submitted to the NCBI.

## Supplementary Materials

Supplementary materials can be found at https://www.mdpi.com/1422-0067/20/17/4151/s1.

## Abbreviations

HIPVs	Herbivore-induced plant volatiles
JA	Jasmonic acid
SA	Salicylic acid
ABA	Abscisic acid
ET	Ethylene
TPS	Terpene synthase genes
*AFS*	*α-Farnesene synthase*
*BGL*	*β-1, 3-Glucanase*
*EF1*	*Encoding elongation factor 1*
*ACS*	*1-Aminocyclopropane-1-carboxylate synthase*
*EIN3*	*Ethylene insensitive protein 3*
FPP	Farnesyl diphosphate
GPP	Geranyl diphosphate
GC-MS	Gas chromatography–mass spectrometry
SPME	Solid phase microextraction
UPLC-QTOF-MS	Ultra–performance liquid chromatography/quadrupole time–of–flight mass spectrometry
