# Supplementary material for: Formation of α-Farnesene in Tea (Camellia sinensis) Leaves Induced by Herbivore-Derived Wounding and Its Effect on Neighboring Tea Plants
Source: Int J Mol Sci. 2019 Aug 25;20(17):4151. doi: 10.3390/ijms20174151 (PMC6747315; doi:10.3390/ijms20174151)
Supplement: Supplementary file 1 [file ijms-20-04151-s001.pdf]

Formation of α-Farnesene in Tea (*Camellia sinensis*) Leaves Induced by Herbivore-Derived Wounding and Its Effect on Neighboring Tea Plants

Authors: Xuewen Wang [1, 2, †], Lanting Zeng [1, 3, †], Yinyin Liao [1, 2], Jianlong Li [4], Jinchi Tang [4], and Ziyin Yang [1, 2, 3]*

Affiliation:

[1] *Key Laboratory of South China Agricultural Plant Molecular Analysis and Genetic Improvement & Guangdong Provincial Key Laboratory of Applied Botany, South China Botanical Garden, Chinese Academy of Sciences, Xingke Road 723, Tianhe District, Guangzhou 510650, China; wangxuewen@scbg.ac.cn (X.W.); zenglanting@scbg.ac.cn (L.Z.); honey_yyliao@scbg.ac.cn (Y.L.)*

[2] *University of Chinese Academy of Sciences, No.19A Yuquan Road, Beijing 100049, China*

[3] *Center of Economic Botany, Core Botanical Gardens, Chinese Academy of Sciences, Xingke Road 723, Tianhe District, Guangzhou 510650, China*

[4] *Tea Research Institute, Guangdong Academy of Agricultural Sciences & Guangdong Provincial Key Laboratory of Tea Plant Resources Innovation and Utilization, Dafeng Road 6, Tianhe District, Guangzhou 510640, China; skylong.41@163.com (J.L.); tangjinchi@126.com (J.T.)*

† *These authors contributed equally to this work.*

* *Correspondence: zyyang@scbg.ac.cn (Z.Y.); Tel.: +86-20-3807-2989 (Z.Y.)*

Supporting information

1. GC-MS analysis

We adopted a GC–MS QP2010 SE (Shimadzu Corporation, Kyoto, Japan) with utilizing GCMS Solution software (Version 2.72, Shimadzu Corporation, Japan) for GC-MS analysis. The column was SUPELCOWAX 10 column (30 m×0.25 mm×0.25 μm, Supelco Inc., Bellefonte, PA, USA). When injecting samples into the GC injection port, here we applied a splitless mode with holding at 230 °C for 1 min. The heating procedure of GC was started from 60 °C for 3 min, then increased to 240 °C at a speed of 4 °C/min and kept at 240 °C for 30 min. Helium was used as the carrier gas with a rate of 1.0 mL/min. We operated full scan mode in analysis of mass spectrometry (mass range, *m/z* 40–200). The α-farnesene authentic standard was used for qualitative and quantitative analyses.

2. UPLC-QTOF-MS analysis

We adopted an UPLC–QTOF–MS (Acquity UPLC I-Class/ Xevo® G2-XS QTOF, Waters Corporation, MA, USA) to analyze samples. The column was Waters ACQUITY UPLC HSS T3 C18 column (2.1 mm×100 mm, 1.8 μm). Milli-Q water with 0.1% (*v/v*) formic acid was Solvent A. Acetonitrile with 0.1% (*v/v*) formic acid was Solvent B. The solvent gradient was started at 20% B, then increased to 35% within 10 min, later increased to 95% B in 0.1 min and kept for 3 min. The flow rate was 0.4 mL/min. The column temperature was 30 °C. The electrospray ionization was operated in negative mode. The MS conditions were capillary voltage: 1.5 kV; source temperature: 100 °C; desolvation temperature: 300 °C; cone gas flow: 50 L/h; and desolvation gas flow: 600 L/h. The quantitative analyses of phytohormones were based on calibration curves, which were constructed by plotting the concentration of each phytohormone against the peak area of the authentic standard.

3. RT-qPCR analysis

We used a Roche LightCycle 480 (Roche Applied Science, Mannheim, Germany) for analysis. The reaction process was one cycle of 95°C for 60 s, 40 cycles of 95 °C for 15 s, and 60 °C for 30 s in a 20 μL reaction system which contained 10 μL of iTaqTM Universal SYBR Green Supermix (Bio-Rad, Hercules, CA, USA), 0.4 μL of each specific forward and reverse primer, 2 μL of cDNA, and 7.2 μL of ddH_2O. A melt curve was performed at the end of each reaction to verify PCR product specificity. The $2^{-\triangle\triangle Ct}$ method was used to calculate the relative expression level. Changes in mRNA level of the test genes for each treatment were normalized to that of *CsEF1*.
